# Effective communication during the COVID-19 pandemic: Social versus traditional media channels

**DOI:** 10.1093/geroni/igaf122.4105

**Published:** 2025-12-31

**Authors:** Reilly Burns, Michele Wood, Pimbucha Rusmevichientong, Jennifer Piazza

**Affiliations:** California State University, Fullerton, Moreno Valley, California, United States; California State University, Fullerton, Fullerton, California, United States; California State University, Fullerton, Fullerton, California, United States; California State University, Fullerton, Fullerton, California, United States

## Abstract

During a public health crisis, it is vital to understand how individuals respond to information regarding potential health risks. The COVID-19 pandemic was an example of such a crisis, where information often conflicted and trust in different sources varied. Understanding which sources improve adherence to established medical guidelines is important, particularly for older adults who are vulnerable to infection. The current study tested whether information communicated through social media (e.g., Facebook, Tik-Tok) versus traditional media sources (e.g., newspapers, television) was differentially associated with actions taken to prevent COVID-19 infection. Participants included 425 Orange County, California residents (Mage: 47.6; range: 18-88), who completed an online survey in July of 2020. Results revealed that people were more likely to receive information on how to prevent COVID-19 infection (e.g., maintaining physical distance, wearing a face covering) from social media sources (b =.127, p = .03) compared to traditional media sources (b =.071, p = .30). Information communicated through social media, however, was unrelated to actions taken to prevent infection (b = -.011, p =  .746) or perceived effectiveness of those actions (b = -.018, p = .632). In contrast, information communicated through traditional media sources was related to more actions taken to prevent COVID-19 infection (b = .129, p = .002) and greater perceived effectiveness of those actions (b = .115, p = .008). Findings indicate that although social media is a primary source of health information, it may not be as effective at eliciting behavioral change as traditional media sources.

